# Effectiveness of a package of postpartum family planning interventions on the uptake of contraceptive methods until twelve months postpartum in Burkina Faso and the Democratic Republic of Congo: the YAM DAABO study protocol

**DOI:** 10.1186/s12913-018-3199-2

**Published:** 2018-06-11

**Authors:** Nguyen Toan Tran, Mary Eluned Gaffield, Armando Seuc, Sihem Landoulsi, Wambi Maurice E. Yamaego, Asa Cuzin-Kihl, Seni Kouanda, Blandine Thieba, Désiré Mashinda, Rachel Yodi, James Kiarie, Suzanne Reier

**Affiliations:** 1 Department of Reproductive Health Research, World Health Organization, Avenue Appia 20, 1211 Genève 27, Switzerland; 2 Institute of Demography and Socioeconomics (IDESO), Geneva University, Boulevard du Pont d’Arve 40, 1211 Geneva, Switzerland; 3 Australian Centre for Public and Population Health Research, Faculty of Health, University of Technology, PO Box 123, Sydney, NSW 2007 Australia; 4 Institut de Recherche en Sciences de la Santé, 03 B.P. 7192, Ouagadougou 03, Burkina Faso; 5 Institut Africain de la Santé Publique, 12 B.P. 199, Ouagadougou, Burkina Faso; 6 School of Public Health, University of Kinshasa, Kinshasa, Democratic Republic of Congo

**Keywords:** Health services operational research, Postpartum family planning, Pregnancy, Antenatal care, Postnatal care, Public health intervention package, Contraceptive uptake, Complex intervention, Qualitative research, Cluster randomized controlled trial

## Abstract

**Background:**

Postpartum family planning (PPFP) information and services can prevent maternal and child morbidity and mortality in low-resource countries, where high unmet need for PPFP remains despite opportunities offered by routine postnatal care visits. This study aims to identify a package of PPFP interventions and determine its effectiveness on the uptake of contraceptive methods during the first year postpartum. We hypothesize that implementing a PPFP intervention package that is designed to strengthen existing antenatal and postnatal care services will result in an increase in contraceptive use.

**Methods:**

This is an operational research project using a complex intervention design with three interacting phases. The pre-formative phase aims to map study sites to establish a sampling frame. The formative phase employs a participatory approach using qualitative methodology to identify barriers and catalysts to PPFP uptake to inform the design of a PPFP intervention package. The intervention phase applies a cluster randomized-controlled trial design based at the primary healthcare level, with the experimental group implementing the PPFP package, and the control group implementing usual care. The primary outcome is modern contraceptive method uptake at twelve months postpartum. Qualitative research is embedded in the intervention phase to understand the operational reasons for success or failure of PPFP services.

**Discussion:**

Designing, testing, and scaling-up effective, affordable, and sustainable health interventions in low-resource countries is critical to address the high unmet need for PPFP. Due to socio-cultural complexities surrounding contraceptive use, this research assumes that this is more effectively accomplished by engaging key stakeholders, including adolescents, women, men, key community members, service providers, and policy-makers. At the individual level, knowledge, attitudes, and behaviors of women and couples toward PPFP will likely be influenced by a set of low-cost interventions. At the health service delivery level, the implementation of this trial will probably require a shift in behavior and accountability of providers regarding the systematic integration of PPFP into their clinical practice, as well as the optimization of health service organization to ensure the availability of competent staff and contraceptive supplies.

**Trial registration:**

Retrospectively registered in the Pan African Clinical Trials Registry (PACTR201609001784334, 27 September 2016).

## Background

Ignoring the contraceptive needs of postpartum women are missed opportunities in health service delivery to ensure that every woman and her family can enjoy the health, social, and economic benefits of family planning (FP).

By spacing the birth-to-pregnancy interval by at least two years and the inter-birth interval by at least three years, the use of effective postpartum contraceptive methods in less developed countries could prevent poor maternal, perinatal, and neonatal health outcomes, including stillbirth, prematurity, low birth weight, neonatal and maternal mortality [1–3]. However, among women in developed countries, the impact of short birth-to-pregnancy intervals is mixed considering recent studies [4–6]. PPFP can be therefore defined as the prevention of unwanted pregnancies and closely spaced pregnancies during the first 12 months after delivery – during this period, pregnancy poses the greatest risks to both the mother and baby [7].

Despite the importance given by the International Conference on Population and Development to FP and timely birth spacing (“*that all couples and individuals have the basic right to determine freely and responsibly the number and spacing of their children and to have the information, education and means to do so”)*, unmet need for FP in the postpartum period remains unacceptably high and far exceeds the FP unmet need of other women. Birth-to-pregnancy intervals in 50% or more of pregnancies in low- and middle-income countries are too short – at less than 23 months [8]. Depending on the definitions of unmet need for PPFP and according to estimates derived from Demographic Health Survey (DHS) data analysis from 57 countries during 2005–2013, the proportion of postpartum women who no longer wanted children or wanted to postpone another child for at least two years but were not using a contraceptive method ranged from 32% (at the end of amenorrhea) to 62% (soon after birth and before the sixth week postpartum; this prospective definition assumes that women should be offered a contraceptive method even while they are still amenorrheic and does not take into account the protection offered by amenorrhea and abstinence) [9]. The unmet need soon after birth even reaches 75% for the West and Central Africa region. The overall goal of our study is to address this high unmet need for PPFP.

Routine reproductive, maternal, newborn and child health (RMNCH) services offer frequent points of contact for providers and women and are key opportunities to address the contraceptive needs of postpartum women and couples [10]. PPFP should therefore be an integral part of RMNCH services. Despite observed advances in access to RMNCH services in many Sub-Saharan African countries in recent decades, progress is weak regarding effective postpartum contraceptive use [8, 11, 12]. There are multiple factors that can be examined from the perspective of the demand side on the one hand (factors related to the demand of health services by women), and supply side on the other (factors related to the provision of health services).

From the demand side: first, abstinence from sex for long periods after childbirth. Second, reliance on amenorrhea are common traditional PPFP methods, especially in Sub-Saharan Africa, where women generally breastfeed their babies up to two years, although not exclusively [13]: among children younger than six months in low- and middle-income countries, 63% are not exclusively breastfed [14]. The length of postpartum amenorrhea depends on the breastfeeding practices. Women often rely on these methods without knowing their limitations. For women who are not breastfeeding, pregnancy can occur within 45 days postpartum even before the return of menses, and among women who are exclusively or almost exclusively breastfeeding, around a quarter (28%) are at risk of pregnancy before six months postpartum [15, 16]. Third, women tend to adopt modern contraceptives only after the resumption of sexual activity or menses [17]. These trends differ between rural and urban areas, as suggested by qualitative research in the urban setting of Ouagadougou, Burkina Faso, where women interviewed did not consider amenorrhea as a protection against pregnancy and all had adopted a contraceptive method or had the intention to do so just before the resumption of sexual activity [18]. Fourth, opposition from partners or others, including for religious reasons, can prevent women from practicing contraception after birth. In West and Central Africa, one in five women reported this barrier [9].

From the supply side: first, contraceptive services may be limited to certain days of the week and only offered once during the six-week postpartum period. Second, service providers sometimes require amenorrheic postpartum women, who missed their 6-week postpartum visit, to wait for the return of their menses or take a pregnancy test before they can receive a contraceptive method.

Several reviews of the literature and of PPFP interventions in less developed countries were recently published [19–22]. The World Health Organization (WHO) and key partners published in 2013 programmatic strategies for PPFP to support program managers in their efforts to integrate PPFP into national and local health strategies [23]. This document reviews the evidence according to the RMNCH continuum as it offers convenient entry points for PPFP interventions: antenatal care (ANC), labor and delivery, postnatal care (PNC), immunization, and child health. The following interventions may have a positive effect on postpartum contraceptive uptake: (1) counseling activities in ANC [24–28]; (2) provision of PPFP information, education, and counseling materials before the woman is discharged home from the health facility, including the provision of emergency contraception backup for lactational amenorrhea method (LAM) users [29–32]; (3) exclusive breastfeeding promotion by community-based counsellors on exclusive breastfeeding practices before five to six months postpartum [33, 34]; (4) access to contraceptive methods (including intrauterine devices (IUD)) immediately after childbirth [35–37]; (5) provider competencies in quality counseling and the provision of quality services with a range of readily available products [38–40]; (6) longer programs with several contact points between providers and clients across the continuum of care versus short ANC interventions [41–43]. While integration of PPFP into immunization and child health services may yield positive results, programmatic challenges (e.g., provider competencies, time pressure, and competition with other priority interventions) and policy barriers remain [44–48]. This said, the evidence is often weak or incomplete and there are still knowledge gaps, particularly regarding studies that explore the desires, intentions, and priorities of women or couples related to PPFP, which may differ across settings, and that look at operationally-feasible ways to integrate PPFP into existing ANC and PNC services. Further, there is a limited number of methodologically rigorous trials that give a detailed description of tested interventions and of how these were implemented.

### Study overall objective and hypothesis

Against the above background and in light of the socio-cultural and health service challenges related to contraception in general and to PPFP in particular, we plan a multipronged and multifaceted operational research intervention: the YAM DAABO study (i.e., “your choice” in Mooré, a national language in Burkina Faso). The overall objective of this study is to determine the effectiveness of a context-sensitive PPFP intervention package on the uptake of contraceptive methods during the first year postpartum compared with the general standard of care. We assume that a package containing different components is more effective than a single intervention to increase contraceptive uptake. The other working hypotheses for the package interventions to be effective and sustainable are as follows: they should (1) strengthen existing ANC and PNC services in primary health care centers; (2) account for the views and preferences of key actors, including those of clients; (3) be in line with national health policies; and (4) take into consideration the scarcity of public resources. Under Methods, we outline preliminary contents of the package.

### Study countries

The study will be implemented in two countries: Burkina Faso (by the Health Sciences Research Institute / Institut de Recherche en Sciences de la Santé (IRSS) in Ouagadougou) and the Democratic Republic of Congo (DRC) (by the School of Public Health / Ecole de Santé Publique (ESP) of Kinshasa University). Table 1 presents relevant reproductive health and health service utilization indicators based on DHS data from Burkina Faso (2010) and the DRC (2013–2014) [49, 50].

**Table 1:** Selected indicators on reproductive health and utilization of maternal and child health services in Burkina Faso (2010) and the Democratic Republic of Congo (DRC) (2013–2014)

	Burkina Faso	DRC
Contraceptive unmet need among postpartum women		
Right after childbirth (%)	89	66
After six months of amenorrhea (%)	50	48
At the end of amenorrhea (%)	24	28
Median duration of breastfeeding (months)	24	22
Exclusive breastfeeding (months)	0.6	2
Median duration of postpartum abstinence (months)	8	8
Median duration of amenorrhea (months)	12	13
Median duration of birth intervals (months)	36	31
Proportion of children born less than 24 months after their previous siblings (%)	13	27
Proportion of women attending antenatal care (%)	95	88
Proportion of deliveries in a health facility (%)	66	80
Proportion on postpartum women receiving prenatal care within 48 h of childbirth (%)	72	44
Proportion of children 12–23 months having received all recommended vaccines (%)	81	41

Sources: [49, 50]

## Methods

### Conceptual framework

To ensure that the PPFP intervention package that will emerge from this trial strengthens the existing ANC and PNC services in primary health care settings, and is effective, safe, sustainable, scalable, and culturally-appropriate, this health services research is grounded by two conceptual frameworks. First, the WHO Health Systems Framework with its six system building blocks (leadership/governance, health care financing, health workforce, medical products and technologies, information and research, and service delivery) [51]. Second, the WHO framework for ensuring human rights in the provision of contraceptive information and services [52] to foster an approach towards optimizing contraceptive choices within the context of repeated visits during routine ANC, postpartum care, and PNC, as recommended by the WHO [53]. This framework comprises the following elements: non-discrimination, availability, accessibility, acceptability, quality, informed-decision making, protection of privacy and confidentiality, participation, and accountability. Both frameworks are interrelated and will inform the design of the PPFP intervention package and the research instruments. For the qualitative and quantitative research phases, the research team will pay attention that no women or adolescent girls will be discriminated against (such as for study participation or in the provision of PPFP information and services), based on their age, marital status, HIV status, membership of a minority, or other medical or social criteria. While ensuring that human rights are upheld in the design and implementation of the study is critical and appears feasible, the researchers are aware that addressing the barriers and catalysts related to the six health system building blocks through the perspective of PPFP is vast and extends well beyond the available study timeframe and resources. For example, the research does not have the objective nor the means to test interventions aimed at reducing health service costs or improving the health information system related to PPFP.

### Phases of the study

**Fig. 1:**
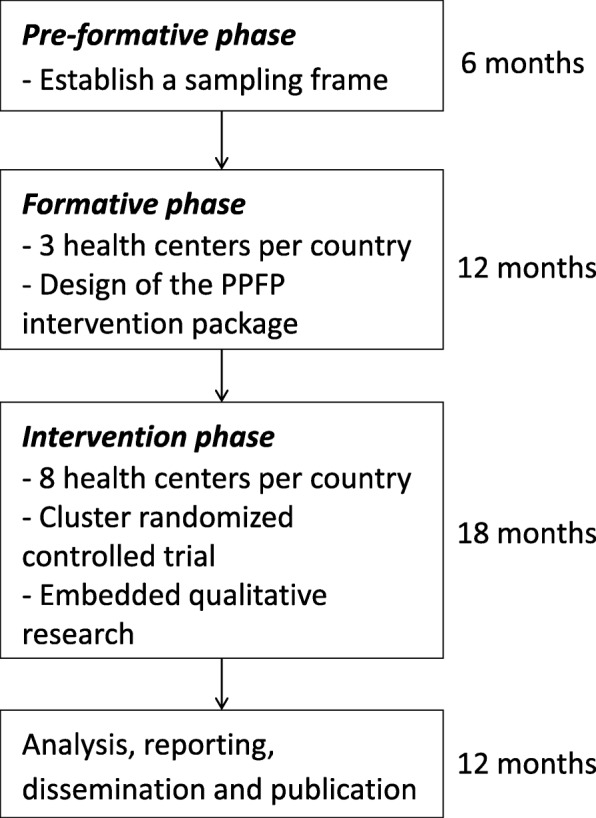
Overall study design

The research entails a complex intervention design with three interacting study phases: a pre-formative phase, a formative phase, and an intervention phase. Figure 1 summarizes the overall study design. These phases are interconnected and have specific objectives, methodologies, and results. Although the proposed methods in each phase are not complex per se, the overall intervention design is complex as it contains “several interacting components”, deals with a “number and difficulty of behaviors required by those delivering or receiving the intervention”, and engages a “number of groups or organizational levels targeted by the intervention”, among others [54]. The design follows the recommendations from the Medical Research Council (2008/2010) to develop a complex intervention, determine its feasibility, implement, and evaluate [54].

The specific objective of the ***pre-formative phase*** is to establish a sampling frame of potential study sites by mapping primary health centers according to pre-established criteria. Health centers are eligible if (1) they offer the continuum of ANC, delivery, and PNC; (2) they provide a selection of at least three modern contraceptive methods, including a barrier method such as condom, a short-term method such as pills, a long-term method such as intra-uterine device and referrals for permanent methods to clients; (3) there were no stock-outs of contraceptives during the preceding six months; (4) they have on average at least thirty deliveries per month; (5) they are situated within four hours drive from the research center; and (6) they are willing to participate. Based on this sampling frame, in each country, three health centers will be chosen conveniently for the formative phase while eight others will be allocated randomly to the experimental and control groups of the intervention phase.

The specific objective of the ***formative phase*** is to identify through participatory action research a context-specific package of PPFP interventions to be implemented during the intervention phase. Initially, with a view to informing the design of the PPFP intervention package, we will seek the participation of a variety of stakeholders to identify barriers and catalysts related to PPFP uptake. Based on field observations from our research teams, service providers did not integrate systematically PPFP into ANC, delivery care or PNC, and there were no dedicated PPFP job aids or counseling tools, although PPFP information and services should be part of the maternal health continuum of care according to national recommendations. To address these knowledge gaps in our study countries (and in other Sub-Saharan countries in general) and to answer providers’ request for a counseling tool to help them better and more systematically support women, we identified the following potential components that would ensure that PPFP is delivered as part of the intervention package: (i) a counseling tool for clients and providers that is specific to PPFP and for use during ANC and PNC services, and (ii) refresher trainings to enhance service providers’ PPFP knowledge and skills. This preliminary set of interventions will be enriched and undergo changes as results of the participatory action research engaging stakeholders are made available. To be integrated into the final package, interventions will need to take into account existing human resources and clinical capacity at the primary health care level, as wells as costs, acceptability, feasibility, and other field considerations.

The specific objective of the ***intervention phase*** is to determine, through a cluster randomized controlled (RCT) trial design, the effect of the PPFP intervention package on the uptake of contraceptive methods during the extended postpartum period, in comparison with the standard of care. The intervention phase also integrates qualitative research that is aimed at identifying operational reasons for success or failure in the implementation of PPFP services.

The phased approach adopted in this study, with the different specific objectives, levels of activities, and intermediate and final results, is underpinned by the theory of change [55]. This theory is helpful in the design of complex interventions such as the one proposed in this trial: it takes as a starting point the ultimate objective of meeting the contraceptive needs of women during the extended postpartum period; it then engages various stakeholders in identifying the desired intermediate results that aim to strengthen ANC and PNC services, before formulating the interventions that are needed to achieve these results. This backward planning allows from the outset a dynamic and participatory approach through which the final PPFP intervention package will be designed. The theory of change will be applied to design quantitative process indicators and qualitative tools aimed at assessing the level of implementation of the intervention package and how each intervention will be implemented. These process indicators and information will be key to help explain why the package is effective or not and highlight operational barriers and enablers useful for future scale-up and implementation research.

We will present the details of the qualitative research and the cluster RCT in the following sections.

### Qualitative research

#### General design

For the qualitative components of the study (in the formative phase and embedded in the intervention phase), we adopt a participatory approach using focus group discussions (FGD) and in-depth interviews (IDI) with members of the community (women, adolescent girls, partners, people with influence), health staff (service providers, managers), and national policymakers.

The ***formative phase*** will take place in each country in three conveniently sampled health centers and their respective coverage areas. These sites are drawn from the sampling frame established in the pre-formative phase.

For the identification of ***PPFP barriers and catalysts***, we plan separate FGDs of six to ten participants for the following categories of individuals: (a) women who are currently pregnant or who delivered at the health center during the previous 12 months – the age will determine their allocation to subgroups: adolescent girls (≤ 19 years), women 20–39 years, women ≥40 years; (b) men (25–40 years) who are partners or husbands of women who are pregnant or who delivered at the health center during the previous 12 months; and (c) service providers. We will conduct IDIs with clinic managers, policymakers, and individuals whom local informants will identify in each recruitment zone as influential, such as religious leaders, village chiefs, community personalities, or others. Adult participants will give their informed consent. Non-adult participants will give their informed assent and their respective parents or guardians their informed consent. The interview guides will cover the following themes related to PPFP: knowledge, attitudes and practices; socio-cultural and health systems barriers and catalysts; and quality of care, including human rights considerations.

We will pilot a draft of the ***PPFP counseling tool*** and finalize it during the formative phase using participatory action research principles. We will engage service providers and clients to design and implement the tool and participate in research to assess its ease of understanding, relevance, contents related to human rights principles, and potential impact. Service providers will be trained on the use of the counseling tool through theoretical sessions and role plays, before implementing it with clients in health centers. These clients will be pregnant women attending ANC services or women who delivered in the previous 12 months. We plan separate FGDs with service providers who offered the counseling tool and with women who received counseling with the tool. If partners attended a counseling session with the tool, they will be invited to a separate FGD to provide feedback.

***Qualitative research embedded in the intervention phase*** will take place at the eight health centers randomized to the experimental or control groups. In line with the theory of change approach, it will consist of an implementation evaluation to analyze barriers and catalysts related to the delivery of PPFP services, assess intervention fidelity (in the experimental group), and explore approaches for scaling up. Separate FGDs are planned for service providers, participants (≤ 19 years, 20–39 years, ≥ 40 years), and their partners. IDIs are planned for clinic managers. The interview guides will cover the following themes related to PPFP service provision: operational barriers and catalysts; quality of care; human rights; and scaling-up considerations.

#### Qualitative data management and analysis

The semi-structured interview guides are in French. In each country, we will recruit a team of social scientists with relevant experience in qualitative methods and in working in the respective local languages (Mooré in Burkina Faso and Lingala in the DRC). We will train them on how to conduct the interviews, paying attention to finding the most appropriate and clear language to express technical terms in the local languages.

The interviews will take place in a location guaranteeing privacy (for example, an empty room in the health facility). The interviews will be audiotaped after obtaining the agreement from participants. Research assistants will transcribe and translate the audio records into French using Microsoft Word. Accuracy checks will be done by comparing transcripts with audio files.

We will perform a thematic analysis using QSR NVivo 11 software, a qualitative research management tool. We will establish a basic codebook, which describes all the nodes, and use it to code data. We will enrich the codebook with new emerging nodes during the coding process. Data will be coded by research assistants. To ensure quality of data coding, we will first ask research assistants to code a common transcription sample before performing inter-rater reliability testing by computing the Cohen’s Kappa coefficient. We will consider a Kappa coefficient of 0.8 and above to be an acceptable concordance threshold.

Results from the formative phase will be presented and discussed during a research workshop with the key members of each country research team, including selected service providers from the formative sites as well as the WHO coordinating team. Country participants will be iteratively invited to express their views on possible interventions when examining the potential effectiveness of possible interventions, as well as acceptability to women and providers, feasibility, “integrability” into the RMNCH continuum, sustainability, and scalability. Through iterative individual voting and discussion processes, we aim to reach a consensus on the final PPFP intervention package.

### Cluster randomized-controlled trial

**Fig. 2:**
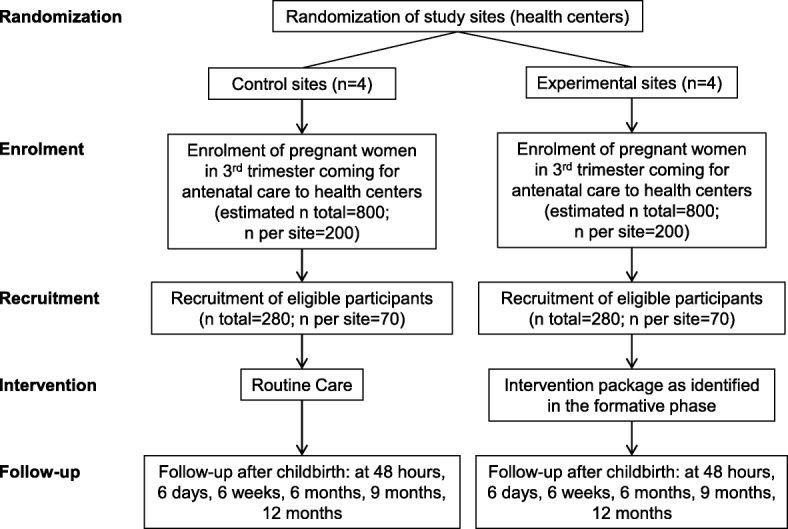
Flow diagram of the cluster randomized control trial in each study country

**Fig. 3:**
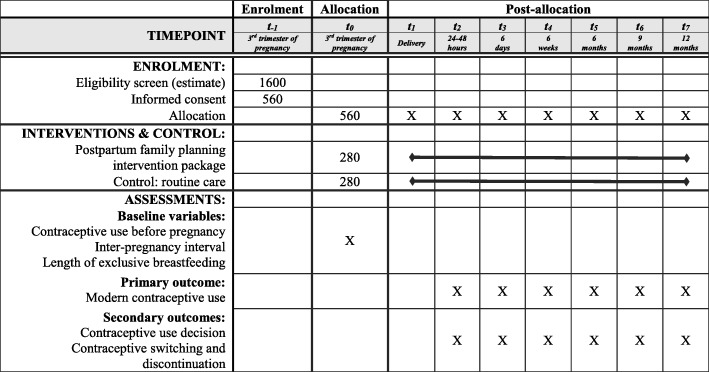
Schedule of enrolment, interventions, and assessments for the cluster randomized controlled trial phase in each study country (SPIRIT template)

This is a two-group, non-blinded cluster RCT conducted in the primary health centers in Burkina Faso and the DRC (see Fig. 2 for the cluster RCT flow diagram, and Fig. 3 for the schedule of enrolment, interventions, and assessments (SPIRIT template)). Randomization to control and intervention arms is at the level of the health center. Participants allocated to the experimental group will receive the PPFP intervention package and those allocated to the control group will receive usual care. Service providers who have midwifery and contraceptive skills and who offer ANC, delivery, and PNC services will implement the clinical components of the PPFP package. The cluster RCT design is justified by the fact that the different components of the PPFP intervention package, which will eventually be identified, may not be delivered directly to individual participants but only applied at the level of the health center (e.g. refresher training for service providers, optimization of health service organization).

#### Outcome measures

Case-report forms (CRFs) will be developed for quantitative indicators. The primary outcome is the uptake of modern contraceptive methods at twelve months postpartum (proportion of women using modern contraceptives at twelve months in the experimental vs. control group).

Secondary outcomes include: use of a modern contraceptive method upon clinic discharge (24–48 h), at six days, six weeks, six months, and nine months postpartum. Intermediate outcomes include the decision taken at different follow-up points to use a contraceptive method. The proposed postpartum follow-up schedule is in line with current national practices in both study countries, although it is slightly different from those recommended by WHO [53]. In 2013, WHO issued the following recommendations for the timing of postnatal visits: on day 1 (24 h), on day 3 (48–72 h), between days 7 and 14, and at week 6.

#### Process measures

It is important to note that the cluster RCT is designed to evaluate the effectiveness of a package of PPFP interventions and cannot indicate which specific intervention in the package will have the most impact on postpartum contraceptive uptake. Therefore, we plan a process evaluation with the objectives to: (1) detect implementation issues that could be explanatory in case the intervention is not effective; and (2) facilitate the replication and scale-up of the intervention if it is proven effective. We have therefore developed process indicators in the CRFs to help assess the level of implementation of selected interventions of the package. Event logs and monitoring visit checklists implemented at health centers will offer other sources of process-oriented information. The qualitative research embedded in the intervention phase will further contribute to understanding the barriers and enablers related to the delivery of PPFP and to the implementation of the trial. The process indicators and the embedded qualitative research will give clues as to the components of the package that participants and providers will perceive as having a greater impact.

#### Sample size calculation

The sample size is estimated based on the following assumptions: among women at six months postpartum, DHS data report 7.2% of use of any method (modern or traditional) in Burkina Faso [56] and 5.1% of use of modern methods in the DRC [10]. These figures allow us to assume that around 5.0% of participants in the control group in both countries will adopt a modern contraceptive at six months postpartum. In Burkina Faso and the DRC, women in the general population have a similar contraceptive prevalence rate for modern methods of 15.0%, which allows us to assume that around 15.0% of participants in the experimental group will use a modern method at six months postpartum. Given the high unmet need for PPFP, we assume that an increased proportion of at least 20.0% of participants in the experimental group will adopt a modern method at twelve months postpartum. This corresponds to a difference of 15.0% between the experimental group (20.0%) and the control group (5.0%). Therefore, assuming an intra-cluster (health center) correlation coefficient of 0.02, we used PASS 11 Software (Power Analysis and Sample Size) for sample size calculation: the experimental group and the control group will each have four study sites with at least 60 participants per site in each study country [57]. This allows for a statistical power of 93% to detect a difference of 15% to a level of significance of 5% at the individual level (and not at the facility level). Assuming a 10% participant loss to follow up, each site will recruit at least 70 pregnant women. This equates to a cohort of 280 pregnant women in each group (4 sites × 70 participants), and in total 560 participants per country (280 participants × 2 study groups). Therefore, across both countries, the study will have a total sample size of 1120 participants (560 in the experimental and 560 in the control group).

#### Eligibility criteria for clusters (health centers) and participants (women)

The study will be conducted in eight primary health centers in each country. The eligibility criteria of health centers were described above under the objective of the pre-formative phase. All pregnant women will be eligible to participate in the study if (1) they are in their third pregnancy trimester; (2) the status of the pregnancy and of the woman allows for a birth at the health center; (3) the woman has the intention to attend ANC, delivery, and PNC at the health center; (4) the woman does not participate in another study; and (5) an informed consent is obtained.

#### Interventions

The health centers of the experimental group will implement the PPFP intervention package. The control group will implement usual care. Depending on the nature of the final package, some interventions may be implemented before participant recruitment (such as PPFP refresher training for service providers), while others will be implemented afterwards (such as using the PPFP counseling tool with participants).

#### Randomization

In each country, the eight sites are matched by pairs according to (1) the average number of deliveries per month; (2) the ratio of health workers per population; and (3) the settings (rural, urban). Within each of the four pairs, we will randomly select the site assigned to the experimental intervention. This randomization is performed four times (once for each pair) in each study country. No restriction in the randomization process is required and masking cannot be done. All consecutive and eligible participants will be included in the clusters.

#### Data source and collection

Data on key outcome measures as well as quantitative process indicators will be collected on paper-based CRFs. The WHO team in Geneva will develop the CRFs with inputs from the country research teams. We will test advanced drafts with an appropriate sample of service providers and mock clients from the formative phase sites before finalizing the forms. Each health center of the cluster RCT will have a research assistant who will be trained to adhere to the study manual and standard operating procedures for data management which are common to both study countries and have been developed by WHO. The information reported on the CRFs will be checked for accuracy and completeness several times at different levels by field coordinators and data managers.

#### Data management

As for other multicenter trials conducted and sponsored by the UNDP/UNFPA/UNICEF/WHO/World Bank Special Programme of Research, Development and Research Training in Human Reproduction (HRP) at WHO in Geneva, we will perform data entry and data quality monitoring using a web-based data management system. The Biostatistics and Data Management team at HRP will develop it through OpenClinica (version 3.11), an electronic data capture software for clinical research, which includes programmed edit checks for data accuracy, completeness, and consistency, reduces delays in data queries and problem resolution, and allows the dataset to be quickly available. The OpenClinica system is a highly secure, open-source, and reliable web-based software solution, which is fully compliant with Good Clinical Practice (GCP) and regulatory guidelines and HRP/WHO Standard Operating Procedures for clinical trial managements.

Data entry will be performed locally in Ouagadougou and Kinshasa by the collaborating research centers and checked by the WHO team in Geneva. We will train the country teams in data entry and data management using the OpenClinica system. Country research teams will be responsible for verifying the data, uploading them onto shared files and entering the data into the OpenClinica system. To reduce errors, double data entry will be performed. The system will automatically generate queries on data (missing values, outliers, inconsistencies, and other errors). The WHO team will monitor data quality. Questions about data inconsistencies or missing values will be sent to sites using standardized forms and will be resolved on an ongoing basis. Data transmission will be encrypted to ensure data integrity and confidentiality of participants. Access to the data will be password protected: only authorized users will be allowed access to the data.

#### Statistical analysis

We will prepare a comprehensive statistical analysis plan before finishing recruitment for the study. The participant will form the unit of analysis and intra-cluster correlation coefficient will be accounted for. All analysis will be by intention to treat. We will perform descriptive statistics by computing means, standard deviations, and minimum and maximum values for continuous variables, and frequencies and percentages for categorical variables. As part of quality control and descriptive analysis of the data, we will examine the distribution of variables to detect outliers. Descriptive statistics will be tabulated for individual clusters and aggregated across clusters. We will use IBM SPSS Statistics 21, R, STATA 13, or SAS statistical packages for the analysis. We will present the results according to the 2010 CONSORT Statement checklist and its extension for cluster studies [58].

#### Intervention fidelity

Threats to the PPFP intervention package fidelity will be minimized by providing an initial training to service providers of the experimental sites and ongoing monitoring and supportive supervision visits to assess adherence to the intervention package. The WHO coordination team will hold weekly teleconferences with each country team and conduct periodic site visits to assess implementation progress. An action plan will be implemented if there are any concerns regarding adherence to the intervention package.

### Ethics

In the design of this trial we have taken into account the recommendations provided by The Ottawa Statement on the Ethical Design and Conduct of Cluster Randomized Trials [59]. This protocol was peer-reviewed by the Research Project Review Panel (RP2) of the HRP. RP2 and the WHO Research Ethics Review Committee, Geneva, Switzerland, approved the study protocol as well as the respective ethical committees in Burkina Faso (see Declarations for details). Upon completion of the formative phase, we will submit to the same global and local ethical committees the amendments to the protocols, which will include the finalized PPFP intervention package and the related research instruments for the experimental phase.

## Discussion

Despite the proven benefits of modern contraceptive use, the unmet need for postpartum family planning remains unacceptably high. This is puzzling as survey findings continue reporting that most couples wish to space their children by at least two years [60], and women access health services more often during pregnancy, delivery, and the first year of their child’s life than other periods. During the multiple opportunities given by this heightened contact with health services, we would expect an increase in demand for and supply of contraceptive information and services.

Given the known reliance on amenorrhea from breastfeeding and abstinence for protection from pregnancy for up to a year postpartum, is it then worth investing energy and resources into PPFP programming during the first year postpartum? Despite these practices, DHS data show that the unmet need for PPFP in Burkina Faso and the DRC, as well as in Sub-Saharan Africa in general, remains critically high and it is important to explore the optimal programmatic approaches to address and resolve it. To this end, different strategies have been explored:

First, there has been a renewed interest on highly effective long-acting and reversible methods, such as immediate postpartum IUD. These programs have shown promising results, although they require major undertaking in terms of logistics, competency training of health staff, and demand creation [37]. Burkina Faso and the DRC have recently given commitments to piloting such programs.

Second, targeted investment to promote LAM for up to six months and switching to another method appears justified from the RMNCH perspective. Tabulations of DHS data on exclusive breastfeeding from Burkina Faso and the DRC are not encouraging and reflect the situation in other low-income countries [61]. The median duration of exclusive breastfeeding changed minimally in Burkina Faso between 1993 and 2010 (0.4 and 0.6 month respectively), and in the DRC between 2007 and 2013–14 (1.4 and 2.2 months respectively). This may be due to widespread early supplementation practices that are entrenched by traditions and hard to influence. The median duration of amenorrhea is twelve months in both countries and suggests that breastfeeding, although not exclusive, must be frequent enough to induce an amenorrheic state. Therefore, having health staff promoting LAM at length may not be an efficient use of limited resources.

Third, with a median duration of postpartum abstinence close to eight months in both study countries, it appears that the immediate postpartum or the six-week postpartum visit dedicated to contraceptive services may not respond to women’s demand. Instead of the targeted “high-dose, low-frequency” strategies that promote a method over another and restrict services to a narrow window period, a “low-dose, high frequency” approach during the end of pregnancy and the first year postpartum to deliver contraceptive information and services may be a more appropriate pathway to respond to the wishes and situations of each postpartum woman and couple: giving information to women and couples, including on benefits and limitations of PPFP and of available contraceptive methods, and letting them choose.

The latter choice-based strategy underpins our health service trial, which also stands out for other reasons. First, given the growing availability of ANC, delivery, and PNC services in primary health centers in low-resource countries, we believe that there is an urgent need to design, test, and scale up effective, affordable, and sustainable PPFP interventions that are not only respectful of the intentions, decisions, and situations of clients but that can also reinforce the existing RMNCH continuum of care. This will allow to minimize missed health services opportunities to address the contraceptive needs of postpartum women and couples. Second, due to the complex social, cultural and religious challenges and opportunities surrounding contraceptive use, our research assumes that this can only be accomplished by following participatory action research principles. Engaged stakeholders include adolescents, women, men, key community members, health staff, policy-makers, and researchers, whose views and inputs will contribute to shaping the PPFP intervention package.

Several changes may happen as a consequence of the study. At the individual level, knowledge, attitudes, and behaviors of women and couples toward PPFP will likely be influenced by a set of inexpensive interventions. At the health service delivery level, implementation of the intervention phase will probably require a shift in behavior and accountability of service providers with regard to the systematic integration of PPFP into their clinical practice, and optimization of health service organization to ensure a reliable availability of competent staff and contraceptive methods and supplies when they are needed.

### Limitations

Foreseen limitations and challenges regarding this complex intervention include coordination difficulties with and between study countries and study sites, political and policy changes, possible time struggle in delivering the intervention and respecting its fidelity, and maintaining the trial timelines. The cluster RCT is not designed to determine which components of the intervention package will have an effect, or not, on contraceptive uptake. To this effect, we have embedded qualitative research and process-oriented indicators in the quantitative research. Although effective in exploring sensitive issues, such as sexuality and contraception, participatory approaches in qualitative research face limitations, including selection bias (e.g., convenience sampling of informants) and response bias (e.g. social desirability, recall bias) – the latter can also affect quantitative measures. To mitigate qualitative research weaknesses, we will sample a sufficient number of participants of different categories to ensure that information saturation can be reached. Findings will also be validated iteratively, including in research workshops that will gather key stakeholders. The overall strengths of the proposed design outweigh its limitations and experienced researchers are joining efforts to form the study team at country and global levels.

### Scaling up

Feasibility and scalability in other low-resource settings are among the criteria that will guide the selection of the final package of interventions. This will ensure that the package in its entirety is widely applicable to and easily scalable in other similar settings. Transparency about the tested interventions and rigorous qualitative and quantitative metrics are paramount in this trial. The results of this trial will be applicable directly to other resource-constrained primary health care settings in Sub-Saharan Africa where RMNCH services are offered.

## Abbreviations

ANC: Antenatal care
CRF: Case report form
DHS: Demographic Health Survey
DRC: Democratic Republic of Congo
FGD: Focus group discussion
FP: Family planning
ICPD: International Conference on Population and Development
IDI: In-depth interview
IUD: Intra-uterine device
LAM: Lactational amenorrhea method
PNC: Postnatal care
PPFP: Postpartum family planning
RCT: Randomized controlled trial
RMNCH: Reproductive, maternal, newborn and child health
UNDP: United Nations Development Programme
UNFPA: United Nations Population Fund
UNICEF: United Nations Children’s Fund
WHO: World Health Organization
